# Using Pooled Local Expert Opinions (PLEO) to Discern Patterns in Sightings of Live and Dead Manatees (*Trichechus senegalensis*, Link 1785) in Lower Sanaga Basin, Cameroon

**DOI:** 10.1371/journal.pone.0128579

**Published:** 2015-07-21

**Authors:** Theodore B. Mayaka, Aristide Takoukam Kamla, Caryn Self-Sullivan

**Affiliations:** 1 Department of Animal Biology, University of Dschang, Dschang, West Region, Cameroon; 2 Oceanographic Center, Nova Southeastern University, 8000 North Ocean Drive, Dania Beach, Florida, 33004, United States of America; University of Western Ontario, CANADA

## Abstract

We aimed at unveiling patterns in live and dead manatee sightings in the Lower Sanaga Basin, Cameroon. For this purpose, the expert opinions of 133 local fishers were collected during in-person interviews, distilled using categorical data analysis, and checked against scientific literature. The five main results are as follows: manatees were sighted averagely once a week in lakes, rivers, and the coast & estuaries, mostly in group sizes of 2-3; the odds of sighting live manatees (respectively dead manatees) decreased (respectively increased) from inland lakes to estuaries and the coast, via rivers; manatee carcasses were reported in all habitats, albeit more frequently in rivers; a distribution map based on fishers’ reports show two manatee concentration areas: Lake Ossa and the Malimba-Mbiako section of River Sanaga; the number of manatees was perceived as increasing despite incidental and directed catches. Thus, our findings corroborate earlier assessments of the Lower Sanaga Basin as being a major manatee conservation area. Additionally, from these results and the literature, we identified three hypotheses about local manatee persistence: deep pools such as lakes offer year round sanctuaries, not just dry-season refugia; seasonality of specific habitat variables determine manatee occurrence patterns; and local variability in habitat encroachment mediate the meta-population dynamics of manatee in the Lower Sanaga Basin. Finally, we examine the implications for data requirements in light of the small ecological scale at which the surveyed fishers ply their trade. Thus, consonant with the Malawi principles for the ecosystem approach to management (www.cbd.int/ecosystem), we recommend collecting data preferably at landscape scale, through a participatory monitoring program that fully integrates scientific and traditional knowledge systems. This program should include, amongst others, a standardised necropsy protocol for collecting mortality and biological data together with sonar and radio-telemetry technology to discern manatee use and movements between critical habitat components.

## Introduction

### Taxonomy, conservation threats and constraints

Manatees (Family Trichechidae; Order Sirenia) and dugongs (*Dugong dugon*, the only extant species of Family Dugondidae; Order Sirenia) are large (adults exceed 3 m and 450 kg), aquatic, herbivorous mammals living in more than eighty tropical and subtropical countries on five continents [1]. The West African manatee (*Trichechus senegalensis*, Link 1795) is the least known of the Trichechidae family, whose other members include the Amazonian manatee (*Trichechus inunguis*) and the West Indian manatee (*Trichechus manatus*) [1, 2]. While the latter two species are vigorously studied (see [3] and references therein), relatively few studies have been published on the West African manatee [4–9]. All three manatee species are classified as vulnerable (*T*. *inungis* and *T*. *senegalensis*) or threatened (*T*. *manatus*), under the IUCN Red List of Threatened Species [1], and listed in Appendix I of CITES (Convention on the International Trade in Endangered Species of Wild Fauna and Flora). Sirenians face similar threats worldwide, albeit at varying degrees [1, 10, 11]. The West African manatee, in particular, may be at greater risk due to hunting pressure and habitat destruction throughout its distribution range, which includes the West African coastal countries from Mauritania to Angola and the inland countries of Mali, Niger, and Chad [11].

The threats that manatees face are many and varied [1, 6–22], but can be grouped conveniently into three broad categories: habitat issues, mortality issues (both related to anthropogenic causes), and stochastic natural events. Habitat loss results from mangrove cutting for the purpose of land development; habitat fragmentation is caused by dam construction; and habitat degradation stems from pollution [1, 9–13, 16–17]. Excessive mortality results from: watercraft collisions (i.e., motorized vessels hitting and injuring or killing manatees, mainly in Florida and Belize), entanglement in fishing gears, and hunting pressure [1, 9–11, 15–16, 18, 22]. Lastly, manatee populations may be under serious stochastic threats as observed in the U.S. Under extreme cold events, manatees may be exposed to very low temperatures for a prolonged period, leading to manatee die-offs [1, 19]. Several microalgae species form harmful algal blooms (i.e. “red tide”) through the release of different biotoxins, causing the death of marine mammals, including sirenians [1, 12, 20–21].

Despite their vulnerable status, manatees continue to be hunted illegally in developing countries, mainly for their meat, skins, oil, bones, tears, and genitals, which are used as food, cooking oil and lamp fuel, folk medicine, crafts, poisons, aphrodisiacs, amulets and charms [1, 6, 16]. In Cameroon alone, 537 interviewees reported 290 kills which, by authors’ account, is an underestimate; furthermore, the highest incidence rate of manatee bycatch was reported from our study area by 3–7% of the interviewed fishers [22]. A wide variety of tools, methods, and systems are used to catch manatees, including: harpoons, nets, box traps, drop traps and artificial feeding stations, baited with cassava and mangrove fruits [1, 6, 16].

**Fig 1:**
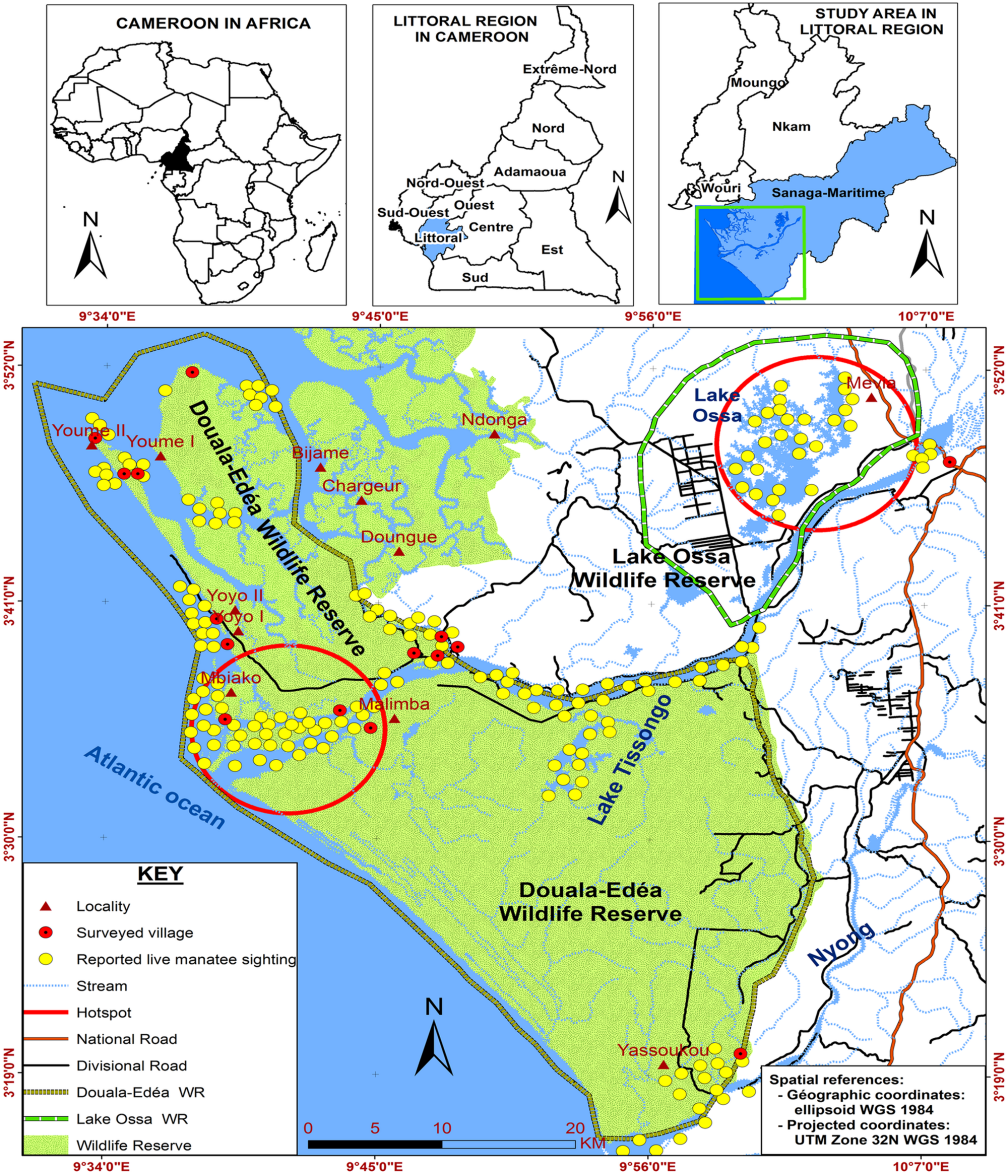
Map of the study area. Surveyed villages are displayed together with the manatee distribution; the two large circles indicate the reported manatee hotspots.

Manatees are very difficult to observe because adequate techniques hardly exist, financial resources are lacking, and because the logistics involved in surveying a cryptic, mildly social species living in murky, little accessible waterways are difficult to organise [23]. For adequate manatee conservation information on species demographics, distribution, habitat availability and suitability is typically lacking. All these constraints prevail in our study area, the Lower Sanaga Basin, a plain stretching from the Atlantic coast of Cameroon over a distance of 100 km landward (Fig 1; see a detailed description in [24]).

### Tapping fishers’ knowledge

Increasingly, ecologists resort to interview-based surveys of local resource users to collect data [13, 22, 24–25]. Resource users hold a wide spectrum of overlapping knowledge of which science and traditional ecological knowledge—synonymous with indigenous knowledge or local knowledge to some extent—are but special cases [26–27]. Once disparaged by Western scientists as anecdotal and non-quantitative, local knowledge is now recognized as valuable to resource management and ecosystem conservation [28–34]. Such knowledge complements science in several important respects. Firstly, it ushers in diachronic observations which, properly marshalled, enable the formulation and testing of scientific hypotheses [30, 32, 35–36]. Secondly, as locality-specific knowledge, local knowledge includes information unknown to (or forgotten by) scientists who infrequently visit remote locations [29, 30, 37]. Thirdly, local knowledge includes information related to taxonomy and systematics [30], population biology [30, 32, 38], and ecology [30, 35, 38, 39].

### Study purpose and scope

This study had a dual purpose. Firstly, we aimed at discerning the patterns in the sightings of live and dead manatees based on fishers’ knowledge. We used the approach known as the pooled local expert opinions (PLEO), as has previously been done with hunters about terrestrial preys [34]. The rationale for using PLEO was that resource users (hunters and fishers) have accumulated invaluable ecological expertise based on a wide range of knowledge forms [26–27]. Secondly, we used the existing literature on manatee biology to substantiate the PLEO and then derive a number of hypotheses that could inform future research in Lower Sanaga Basin.

We focused on fishers’ expert opinions rather than the actual knowledge forms on which these opinions are based. Indeed, while interesting in themselves, such knowledge forms usually carry different connotations for different persons (see, e.g. endnote 1 in [28]). The differences in connotations may actually distract from the central issue, namely the expertise that fishers, whether indigenous or not, have developed of the manatee ecology in our study area. The latter encompasses the Douala-Edéa and Lake Ossa Wildlife Reserves. Because many references concentrate on manatee conservation [1, 9, 13, 16, 24, 40–43], this issue will only be considered incidentally in this paper.

We were able to achieve our objectives within the conceptual, methodological and spatial scope just mentioned, allowing for the obvious limitations inherent to traditional ecological knowledge, namely the small ecological scale at which the surveyed fishers ply their trade. Thus, consonant with the Malawi principles for the ecosystem approach to management (www.cbd.int/ecosystem), we recommend the collection of data at landscape (or regional) scale through a monitoring program that fully integrates the scientific and traditional knowledge systems.

### Research questions and hypotheses

We posed three main questions, matching hypotheses that were formulated based on manatee literature, field observances or conservation requirements. Firstly, we asked whether the pattern in the sightings of live manatees was strongly associated with habitat type (H), time of day (TOD), and season (S). The hypotheses for this question are as follows, *H*_0_ the pattern in the sighting of live manatees is not associated with either habitat type (*H*), time of day (*TOD*), or season (*S*) nor is it associated with any interaction of these classificatory factors (i.e., *H*, *TOD*, *H S*, *S TOD*, and *H TOD S*) versus *H*_1_: the pattern in the sighting of live manatees is significantly associated with at least one of the classificatory factors. Both West Indian and African manatees occur in fresh, brackish, and salty water systems [1, 9, 13, 44]; we expected, however, manatee occurrence to decrease with the salinity gradient. Whereas manatees lack marked diel activity patterns due to the absence of a pinal organ in the brain [1], we expected the manatees to exhibit crepuscular to nocturnal activity patterns in response to increased human pressure [11, 45–47]. Finally, manatees were expected to be sighted less frequently in the dry season than in the wet season because of high water levels and abundant vegetation [6, 44, 48]. The opposite may seem obvious since in the wet season, water turbidity is higher and clarity is poorer, but these factors may not affect the visibility from boats as explained elsewhere (see [49]: p.175).

Secondly, we asked whether the number of sighted dead manatees (D) was strongly associated with habitat type (H) on the one hand and, as a proxy of manatee population size, which in our case is the number of live manatees sighted at once (L) on the other hand. The hypotheses for this question were as follows: *H*_0_: the number of dead manatees sighted (D) is not associated with either habitat type (H), the number of live manatees sighted at once (L), nor is it associated with any interaction of these classificatory factors (i.e. *H D*, *H L*, *D L*, and *H D L*) versus *H*_1_ the number of sighted dead manatees is significantly associated with at least one of the classificatory factors or any interaction thereof. This hypothesis emerged from the necessity to improve the protection of manatee and its habitat. It was therefore important to discern habitats in which manatees were prone to excess mortality, regardless of the causes involved. Furthermore, we wanted to assess the strength of the association between the mortality prevalence and the incidence of live manatee occurrence, so that statistical prediction could be used to inform protection efforts.

Thirdly, we asked fishers to what extent they thought that a change in manatee numbers had occurred over time and habitat types. This query is central to any sound ecological monitoring program aimed at tracking key population features for endangered species, using trained local observers [50–52]. The hypotheses for the third question were as follows: *H*_0_: the proportion of fishers who perceive an increasing or stable trend in manatee numbers is the same in all habitats versus *H*_1_: the proportion of fishers who perceive an increasing or stable trend in manatee numbers differs with habitat type.

## Methods

### Ethics statement

We did not obtain either an approval or a waiver from the National Ethics Committee, as it deals primarily with medical research. However, all ethics and principles of responsible research were observed at all investigation stages. We secured the necessary permits and authorizations for data collection through locally active environmental NGOs (IUCN, Cameroon Wildlife Conservation Society, and Water Task Group), we informed all pertinent stakeholders (fishers, local traditional authorities, representatives of public services and park administration) about the study objectives, and we elicited their consented participation in the interviews. From our side, we fully protected their privacy rights during data recording and storage processes, and we shared the research results (data and reports) fully and openly with each separate group of participants during several evaluation sessions. Finally, in compliance with data access policy, raw data (set out in contingency table format) and the related statistical results appear in the supporting information (S1–S8 Tables and S1–S2 Figs). Further details can be obtained from the lead author upon request.

### Data collection

A stratified random sample of 133 fishers was drawn from four major habitat types (lakes, rivers, coast, and estuaries) for in-person, semi-structured interviews. The questionnaire used for this purpose comprised five sections: fishers identification by name, age, village, and marital status, plus their brief description of manatee, if they claimed to know it; contributed knowledge of manatee ecology: how often and under which circumstances (i.e., location, time of day, season, and group size) manatees were sighted most; report on observed manatee deaths and/or carcasses (frequency, location, and possible causes); enumeration of known manatee diet items and how such knowledge was gained; and finally fisher-manatee conflicts assessment and proposed solutions. The last two sections are not reported in this article. Table 1 gives a synopsis of the questionnaire (types of questions and answers).

**Table 1:** Questionnaire synopsis showing types of questions and answers.

Section^†^	Questions	Answers
I	Age, ethnic group, village?	Integers (19–89); various answers
	Do you know the manatee? If yes, please briefly describe it.	Yes/No; various answers;
	What is your main occupation?	Fisher, oyster gatherer, farmer, other;
	Professional experience (in years);	<1, 1–2, 3–5, 6–10, >10;
	In which season do you fish most?	Dry, Rainy, Any;
II	Number of manatee sightings in a month? Where (village) precisely?	<1, 1–3, 4–6, > 6; Various locations;
	Season you sight manatee most?	Dry, rainy, any
	At which time of day mostly?	Morning 05–11, midday 11–14, afternoon 14–18, evening 18–21;
	At which tide mostly?	High, low, any tide;
	How many manatees sighted at once, averagely?	1, 2–3, 4–8, > 8;
	How manatee numbers have evolved in last decade?	Reduced, constant, increased, no opinion;
	Cause behind this trend?	Excess fishing/hunting, accident, absence of hunting, high reproduction;
III	How many dead manatees ever sighted? Where?	Never, 1–3, 4–6, > 6; Various locations.
	How many carcasses seen at once?	Only one, 2, 3–4, 5–6, > 6;
	Most probable cause of death?	Hunting, collision with boat, captured in net, old age, food intoxication, other;

^†^ The sections pertaining on manatee diet (IV) and fisher-manatee conflicts (V) were not included in the present paper.

The research team, comprising a junior researcher (ATK) escorted by a field assistant and a guide (also interpreter when the need arose), completed each questionnaire within 15 to 20 minutes. A mix of tactics served for bias control, in particular [25]: avoid asking leading questions or/and restricting the number of response choices; prefer closed-format questions over open questions, whenever possible; keep the number of “non-response” cases to a minimum; repeat some questions asked 5–10 minutes earlier; and finally ask a pair of causally linked questions and assess answers for congruence. We fully informed interviewees that they consented to participate in a purely academic exercise and that we protected their confidentiality [22]. These ethical obligations further contributed to lower response bias by allaying any fear of reprisal. Incidentally, we did not attempt to identify manatee hunters among the interviewees, purposely to keep the rate of biased responses low.

### Data analysis

We used software R, version 3.0.2 [53], to implement categorical data analysis [54–55]. Specifically, we used log-linear Poisson models to discern the patterns in sightings of live and dead manatees (research questions 1 and 2). We also used a logistic binary regression to model the proportion of respondents who perceived either an increasing or a stable trend in manatee numbers (research question 3). Both model types are well expounded in statistical textbooks [54–55]. A description of data modeling techniques, model assumptions and bias reduction approaches, and hypotheses testing procedures now follows.

#### Log-linear Poisson models for sightings of live and dead manatees

A log-linear Poisson model served to predict the cell counts in three-way contingency tables. The frequencies of live or dead manatee sightings were crossed-tabulated by three factors only, due to the modest sample size (n = 133). Starting with live manatee sighting, the classificatory factors with their abbreviations and levels are as follows: H for habitat (lakes, rivers, and coast & estuaries); TOD for time of day (morning 05:00–11:00, midday/afternoon 11:00–18:00, and evening 18:00–21:00); and S for season (dry and rainy). The full log-linear model is a counterpart to a three-way ANOVA for categorical data which predicts the frequency in cell (i, j, k) as:

$$\text{log}\text{(}\lambda_{ijk}\text{)}=\lambda +\lambda_{i}^{H}+\lambda_{j}^{TOD}+\lambda_{k}^{S}+\lambda_{ij}^{H\cdot TOD}+\lambda_{ik}^{H\cdot S}+\lambda_{jk}^{TOD\cdot S}+\lambda_{ijk}^{H\cdot TOD\cdot S}$$ (1)

where, *λ*_*ijk*_ is the expected sighting frequency in cell (i, j, k); *λ* is the overall effect; ${\;\lambda }_{i}^{H},\;\lambda_{j}^{TOD},\;$ and $\lambda_{k}^{S}$ are respectively the marginal effects of habitat i (i = 1,2,3), time of day j (j = 1,2,3) and season k (k = 1, 2); $\lambda_{ij}^{H∙TOD},\;\lambda_{ik}^{H∙S},$ and $\lambda_{jk}^{TOD∙S}$ are two-factor interaction (or association) effects of habitat, time of day, and season, taken two at a time; and $\lambda_{ijk}^{H∙TOD∙S}\;$ are the interaction (or association) effects of all three factors taken together.

A similar model applies for the pattern in sightings of dead manatees, using the following classificatory factors: H for the habitat to which the respondents are affiliated (defined as above); D for number of dead manatees ever sighted by respondents (0, 1–2, and 3+); and L for the group size of live manatees sighted at once (1, 2–3, 4+). Using this notation and denoting the model effects by *μ* (instead of *λ*, in order to avoid confusion), the log-linear model for the pattern of sighting dead manatees is expressed as follows:

$$\text{log}\text{(}\mu_{ijk}\text{)}=\mu +\mu_{i}^{H}+\mu_{j}^{D}+\mu_{k}^{L}+\mu_{ij}^{H\cdot D}+\mu_{ik}^{H\cdot L}+\mu_{jk}^{D\cdot L}+\mu_{ijk}^{H\cdot D\cdot L}$$ (2)

where all the terms are defined analogously to those in Eq 1.

The models in Eqs 1 and 2 are both full and saturated, i.e. neither provides an independent estimate of its residual deviance. Also, each model in Eqs 1 and 2 above has eight possible sub-models, of which the best contender was chosen using two quality- of- fit criteria. Firstly, we use the Akaike’s Information Criterion (AIC) to identify the parsimonious sub-model (i.e. the sub-model with the minimum number of parameters). Secondly, we used the chi-square approximation of the likelihood ratio tests (LRT) to find out which sub-model resulted in the most significant reduction of the null deviance [54–55].

The effects in the above log-linear Poisson models are straightforward to understand and to display using plain English; however, the corresponding parameters have a slightly involved notation. Therefore we use the following rule of thumb for easy reference. A parameter symbol, *λ* (patterns in sighting live manatees) or *μ* (patterns in sighting dead manatees), will be superscripted with the factor abbreviations and subscripted with the levels or categories of the corresponding factors. By way of illustration: $\lambda_{mid}^{TOD}$ is the main effect of the midday-afternoon level of the time of day factor on the sighting pattern of live manatee;$\;\lambda_{lake,\;eve}^{H∙TOD}$ is the interaction (or association) effect between the lake level of habitat type and the evening level of the time of day factor on the pattern of sighting live manatee; $\mu_{3+,\;4+}^{D∙L}$ is the interaction (or association) effect between the sighting of three or more dead manatees and the sighting of four or more live manatees on the pattern of sighting dead manatees.

#### Logistic binary model for the perceived increase in manatee numbers

A binary response variable with values 0 or 1 was recorded for each surveyed fisher depending on whether they perceived the manatee numbers as either decreasing or increasing/stable, respectively. Such variables are known to have a Bernoulli rather than a Gaussian distribution [54–55]. Therefore, we used the logistic regression model below to predict in each habitat type the proportion of respondents who perceived the manatee numbers as either increasing or stable:

$$\text{log}[p/\text{(}1-p\text{)}]=\beta_{0}+\beta_{1}R+\beta_{2}C$$ (3)

where *η* (*p*) = log[*p*/(1-*p*)] is is the log-odds of the proportion *p*;*R* and *C* are indicator variables (i.e. with values 1 and 0) respectively for rivers and coast & estuaries. Lake Ossa has been considered to be a manatee sanctuary [6]; therefore, by extension, the lakes in our study area were taken in the model above as benchmarks for gauging other habitat types. Habitat effects are given by the regression coefficients *β*_1_ (rivers) and *β*_2_ (coast &estuaries) adjusting for the intercept, *β*_0_ (lakes, the reference level).

A similar model as in Eq 3 served in predicting the proportions of responses to other binary items, including live manatee sighting and occurrence of incidental catches. The logistic regression provides an exact method for testing the proportion of a binomial variable, unlike chi-square and normal approximation tests [54–55]. The estimated proportions are therefore readily obtained as $\hat{p}=\text{exp}\text{(}\hat{\eta }\text{)}/\text{(}1+\text{exp}\text{(}\hat{\eta }\text{)}\text{)}$ where $\hat{\eta }\;=\;{\hat{\beta }}_{0}+{\hat{\beta }}_{1}R+{\hat{\beta }}_{2}C\;$ is the fitted regression model. Alternatively, the odds-ratio in the lakes (reference level) is $\hat{p}/\text{(}1-\hat{p}\text{)}\;=\;\text{exp}\text{(}{\hat{\beta }}_{0}\text{)},$ which changes to $\text{exp}\text{(}{\hat{\beta }}_{0}+{\hat{\beta }}_{1}\text{)}\;=\;\text{exp}\text{(}{\hat{\beta }}_{0}\text{)}\times \text{exp}\text{(}{\hat{\beta }}_{1}\text{)}$ in the rivers and $\;\text{exp}\text{(}{\hat{\beta }}_{0}+{\hat{\beta }}_{2}\text{)}\;=\;\text{exp}\text{(}{\hat{\beta }}_{0}\text{)}\times \text{exp}\text{(}{\hat{\beta }}_{1}\text{)}$ in the estuaries and along the Atlantic coast. Thus, with respects to the odds in Lake Ossa, the odds multipliers in the last two habitats are respectively $\;\text{exp}\text{(}{\hat{\beta }}_{1}\text{)}$ and $exp\text{(}{\hat{\beta }}_{2}\text{)}$.

#### Model assumptions and bias reduction approaches

As with the Pearson’s chi-square, the log-linear Poisson model requires that there be at least 5 observations by cell [56]. However, the sparseness (number of small/empty cells) increases with the size of a multi-way contingency table, leading to severely biased odds ratio estimates and a poor chi-square approximation of the sampling distribution of fit statistics. Thus, to the extent possible, we heeded the working rule that no cell expectation should be below 1 and that two extreme expectations could be close to 1 provided that most other expectations well exceed 5 (see [56]: p.77). In order to meet the minimum cell requirement, we pooled habitat types and/or response categories (i.e. coast and estuaries, increasing and stable trends in manatee numbers) and we omitted skewed categories (i.e., manatees being sighted at anytime or/and in both seasons) from the statistical analysis. Moreover, for the purpose of bias mitigation, the selected log-linear Poisson (sub) models were refitted to the contingency table using a penalized (i.e. bias-reduced) maximum likelihood procedure proposed by Firth [57] and implemented with a program script available in Kosmidis and Firth [58].

#### Hypotheses testing procedures

The research hypotheses stated earlier were tested after proper reformulation in terms of the statistical model parameters. Firstly, using Eq 1 above for the patterns in the sighting of live manatees, $H_{0}:$ all main and interaction effects are equal to zero, i.e. *log*(*λ*_*ijk*_) = *λ* for all cells (i, j, k) versus *H*_1_: at least one of the main effects $\lambda_{i}^{H},\;{\;\lambda }_{j}^{TOD},{\;\lambda }_{k}^{S}\;$ or interaction terms $\lambda_{ij}^{H∙TOD},\;\lambda_{ik}^{H∙S},{\;\lambda }_{jk}^{TOD∙S}\;$ is significantly different from zero. Secondly, using Eq 2 above for the patterns in the sighting of dead manatees, *H*_0_: all main and interaction effects are equal to zero, i.e. $log\text{(}\mu_{ijk}\text{)}\;=\;\mu \;$ for all cells (i, j, k) versus *H*_1_: at least one of the main effects $\mu_{i}^{H},\;\mu_{j}^{TOD},\mu_{k\;}^{S}$ or interaction terms $\mu_{ij}^{H∙TOD},{\;\mu }_{ik}^{H∙S},{\;\mu }_{jk}^{TOD∙S}$ is significantly different from zero. Thirdly, using Eq 3 above for the perceived increase in manatee numbers, *H*_0_ the regression coefficients *β*_1_ and *β*_2_ are equal to zero versus *H*_1_: at least one of regression coefficients *β*_1_ and *β*_2_ is significantly different from zero.

The precision attached to the estimated model parameters will systematically refer to the estimated standard errors (SE), unless stated otherwise. Thus, the null hypothesis of the general form *H*_0_: *θ* = *θ*_0_ would be rejected in favour of the alternative hypothesis *H*_1_: *θ* ≠ *θ*_0_ if, in absolute value, the Wald’s statistic $\text{(}\hat{\theta }-\theta_{0}\text{)}/SE\text{(}\hat{\theta }\text{)}$ exceeds 1.96 or, equivalently, if the 95% confidence interval $\hat{\theta }\pm 1.96∙SE\text{(}\hat{\theta }\text{)}$ does not contain the value *θ*_0_.

Finally, we used the Pearson’s chi-square (*χ* ^2^) and, when necessary, its likelihood ratio approximation (*G* ^2^) to perform several subsidiary tests, including homogeneity and independence tests [56] as well as model selection tests [54–55].

## Results

### Patterns in the sightings of live manatees

**Fig 2:**
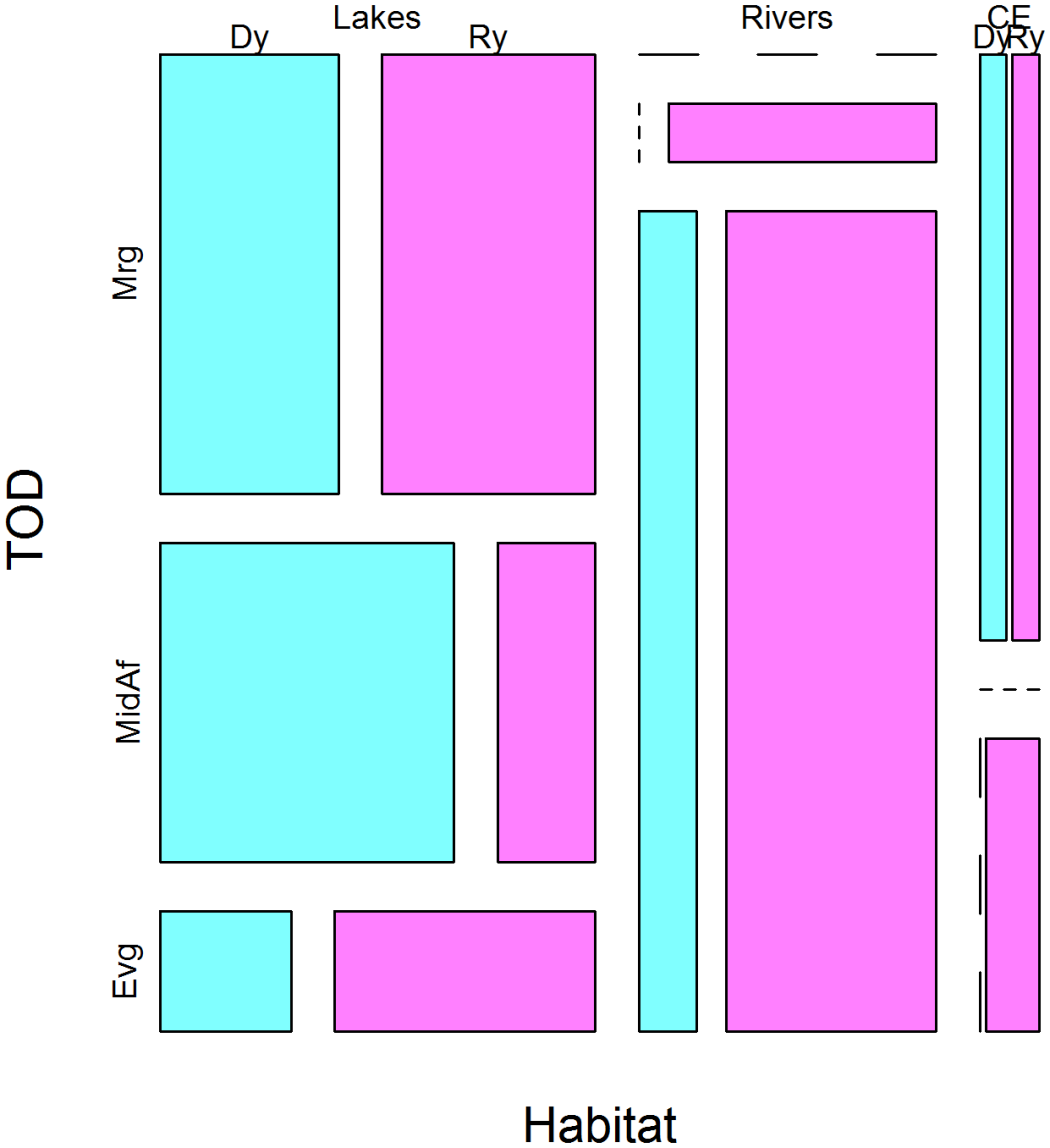
Mosaic plot of patterns in the sightings of live manatees. Live manatee sightings in rivers and coast & estuaries are most frequent in the evenings of the rainy season (Ry). In lakes, however, manatees seem to have a diurnal activity pattern in the morning of both seasons (Dy and Ry) and the midday-to- afternoon period of the dry season (Dy) as opposed to crepuscular-to-nocturnal activity pattern in the rainy season (Ry).

The frequency of sighting live manatees is highest in the rivers during the evening of the rainy season as evidenced in the mosaic plot (Fig 2) and confirmed from the model output in Table 2 (${\hat{\lambda }}_{riv,\;eve}^{H.TOD}\;=\;4.557\pm 1.537,\;Z\;=\;2.964,\;P<0.05$). In the lakes, sightings of live manatees are mostly diurnal: either in the morning of dry and rainy seasons, or else from midday to afternoon in the dry season, as is obvious from the mosaic plot and the highly significant intercept ($\hat{\lambda }\;=\;1.710\pm 0.401,\;Z\;=\;4.260,\;P<0.001$).

**Table 2:** Fitted log-linear Poisson model for patterns in the sightings of live manatees.

Model effects	Estimates	SE	Z-statistics
Intercept	1.709	0.401	4.259***
Rivers	-3.135	1.420	-2.208*
Coast & estuaries	-1.526	0.686	-2.224*
Rainy	0.143	0.518	0.276
Midday/afternoon	0.047	0.568	0.082
Evening	-1.871	0.775	-2.414*
Rivers x Midday/afternoon	1.401	1.663	0.842
Coast & estuaries x Midday/afternoon	-1.307	1.579	-0.828
Rivers x Evening	4.557	1.537	2.964**
Coast & estuaries x Evening	0.679	1.183	0.574
Rainy x Midday/afternoon	-0.762	0.817	-0.933
Rainy x Evening	1.027	0.738	1.392
**Fit statistics** ^†^	**AIC**: NONE		
	**Null deviance**: 60.11 (17 df)		
	**Res. deviance**: 6.21 (6 df)		

The parameter estimates, standard errors, Wald’s Z-statistics and fit statistics were obtained using a penalized (reduced-bias) maximum likelihood (see details in Firth [57] and Kosmidis and Firth [58]).

*,**,*** significant at probability levels 5%, 1%, and 0.1%, respectively.

^†^ The R script used in implementing the penalized maximum likelihood does not return a value for the Akaike’s Information Criterion (AIC).

**Fig 3:**
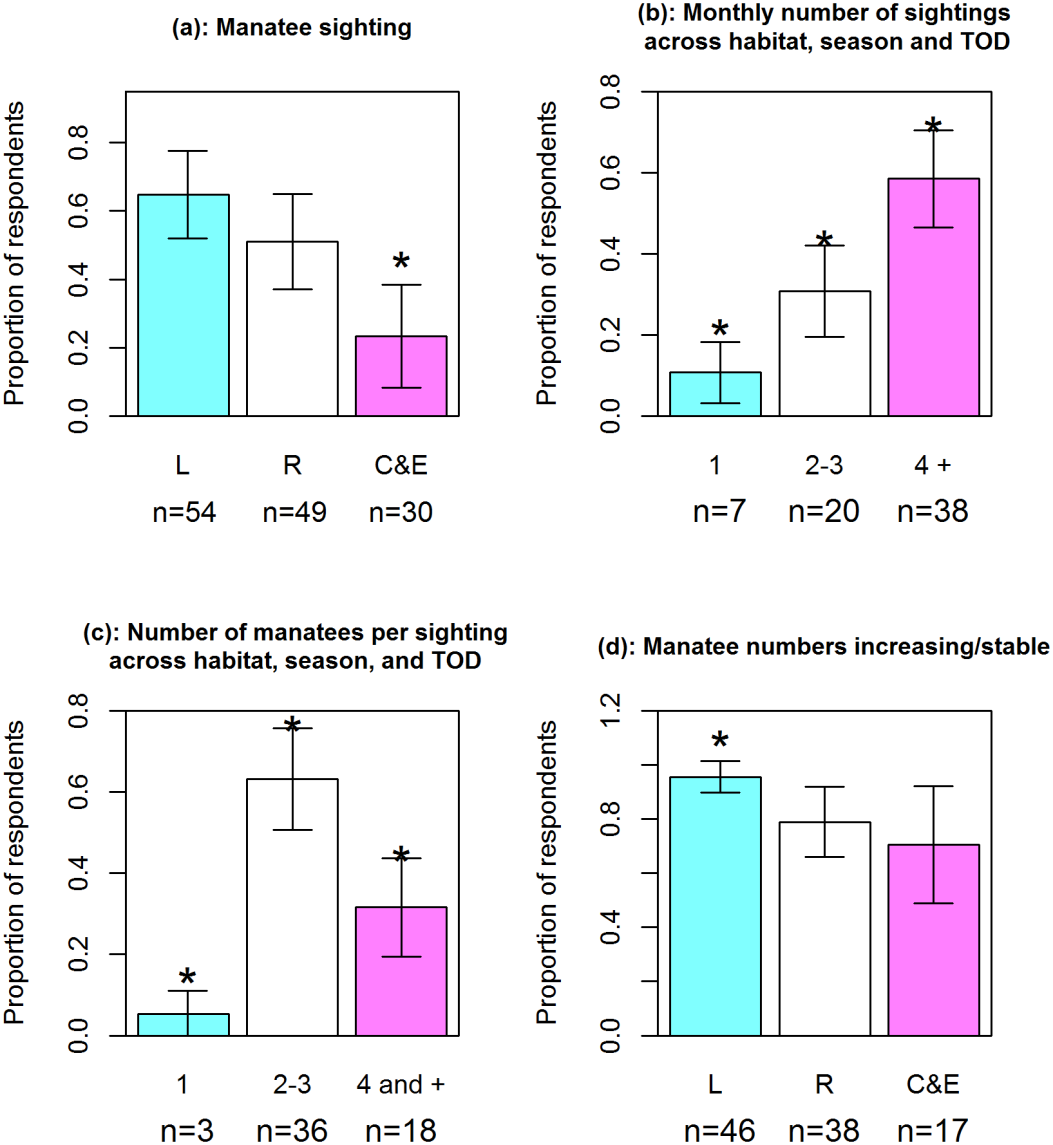
Manatee sighting and occurrence patterns in lakes (L), rivers (R), and coast & estuaries (C&E) . Items concerned are: (a) the proportion of respondents doing the sighting; (b) the monthly number of live sightings; (c) the number of manatees per sighting; and (d) the percentage of perceived increasing/stable trend in manatee numbers. The error bars represent the 95% confidence intervals together with the corresponding sample sizes (shown at panel bottom). An asterisk indicates effects that are significantly different at probability level of 5% or less.

Further insight into the patterns of live manatee sighting was achieved independently of the log-linear Poisson model (see output in Table 3). In particular, the incidence of live manatee sightings is significantly higher in the lakes than in the rivers and the coast & estuaries (*χ* ^2^ = 13.3, df = 2, p<0.01, item 1 in S1 Table), as can be seen further in Fig 3a. In fact, the odds of sighting a live manatee are 1.84 in the lakes compared to 1.04 in the rivers and 0.30 in the coast & estuaries (Table 3). The monthly number of live manatee sightings did not differ significantly with either habitat (*χ* ^2^ = 6.42, df = 4, p = 0.17; item 2 in S1 Table), season (*χ* ^2^ = 5.00, df = 2, p = 0.082) or time of day (*χ* ^2^ = 7.77, df = 4, p = 0.10). Ignoring thus all three classificatory factors (habitats, seasons, and TOD), the monthly frequencies of live manatee sighting decreased as follows: four or more (0.58 ± 0.120), two to three (0.31 ± 0.112) and once (0.11 ± 0.075), whence an overall significant chi-square test (*χ* ^2^ = 22.37, 2*df*, *p* < 0.0001) as can be seen in Fig 3b.

**Table 3:** Fitted logistic regression models for binary response variables^§^.

Model effects^†^	Proportion of respondents sighting live manatees		Proportion of respondents reporting incidental catches		Proportion of respondents perceiving increasing/stable manatee numbers	
	*Estimate (SE)*	*Odds-ratio*	*Estimate (SE)*	*Odds-ratio*	*Estimate (SE)*	*Odds-ratio*
Lakes	0.61(0.29)*	1.84	-2.92(0.73) ***	0.05	3.09(0.72)***	22.00
Rivers	-0.57(0.40)^ns^	0.57	0.43(0.94)^ns^	1.54	-1.77(0.83)*	0.17
Coast & Estuaries	-1.80(0.52)**	0.17	2.33(0.84)**	9.25	-2.22(0.90)*	0.11
**Fit statistics**						
*Null deviance (df)*	184.37(132)		77.83(101)		84.87 (100)	
*Residual deviance (df)*	170.55(130)		64.8(99)		76.16(98)	
*LRT(2df)* ^‡^	13.82 (2) **		10.35(2)**		82.16(2)**	
*AIC*	176.55		73.48		82.16	

^§^The levels of significance of parameter estimates (with standard errors in parentheses) are as follows: n.s. for not statistically significant;

*, **, and *** for significant at 0.05, 0.01, and 0.001 probability levels, respectively.

^†^ The logistic regression is given as ln[*p*/(1-*p*)] = *β*_0_ + *β*_1_R + *β*_1_ *C* (Eq 3 in text body), the predictors R and C being indicator variables (with values 1 and 0) respectively for rivers and coast & estuaries. The regressions coefficients *β*_1_ (rivers) and *β*_2_ (coast & estuaries) are adjusted for the intercept, *β*_0_ (lakes, the reference level). The estimated odds ratio $\hat{p}/\text{(}1-\hat{p}\text{)}\;=\;exp\text{(}{\hat{\beta }}_{0}+{\hat{\beta }}_{1}R+{\hat{\beta }}_{1}C\text{)}$ is equal to exp(${\hat{\beta }}_{0}$) in the lakes, $exp\text{(}{\hat{\beta }}_{0}+{\hat{\beta }}_{1}\text{)}$ in the rivers and $exp\text{(}{\hat{\beta }}_{0}+{\hat{\beta }}_{1}\text{)}$ in the coast & estuaries.

^‡^ LRT = Likelihood ratio test for the model overall significance approximates a chi-square test as the difference between null deviance (intercept only) minus residual deviance (intercept plus the regression coefficients) with two degrees of freedom (number of additional model parameters).

Using the same sequence of statistical tests as above, we found that the number of manatees per sighting did not change with habitat (*χ* ^2^ = 1.64, 4 df, p = 0.80, item 3 in S1 Table), season (*χ* ^2^ = 0.1595, 2 df, p = 0.92) or time of day (*χ* ^2^ = 0.68, 4 df, *p* = 0.95). Ignoring once again all three classificatory factors, two-to-three manatees were more often sighted (0.63±0.125) than four manatees and plus (0.32±0.121) or a single manatee (0.05±0.058) as seen in Fig 3c and statistically confirmed (*χ* ^2^ = 28.74, 2 df, p <0.0001).

A map of manatee distribution was drawn based on fishers’ reports. There seems to be two concentration areas: one in Lake Ossa, and the other in the Malimba—Mbiako section of River Sanaga, towards the estuary (shown with the red circles in Fig 1).

### Patterns in the sightings of dead manatees

**Fig 4:**
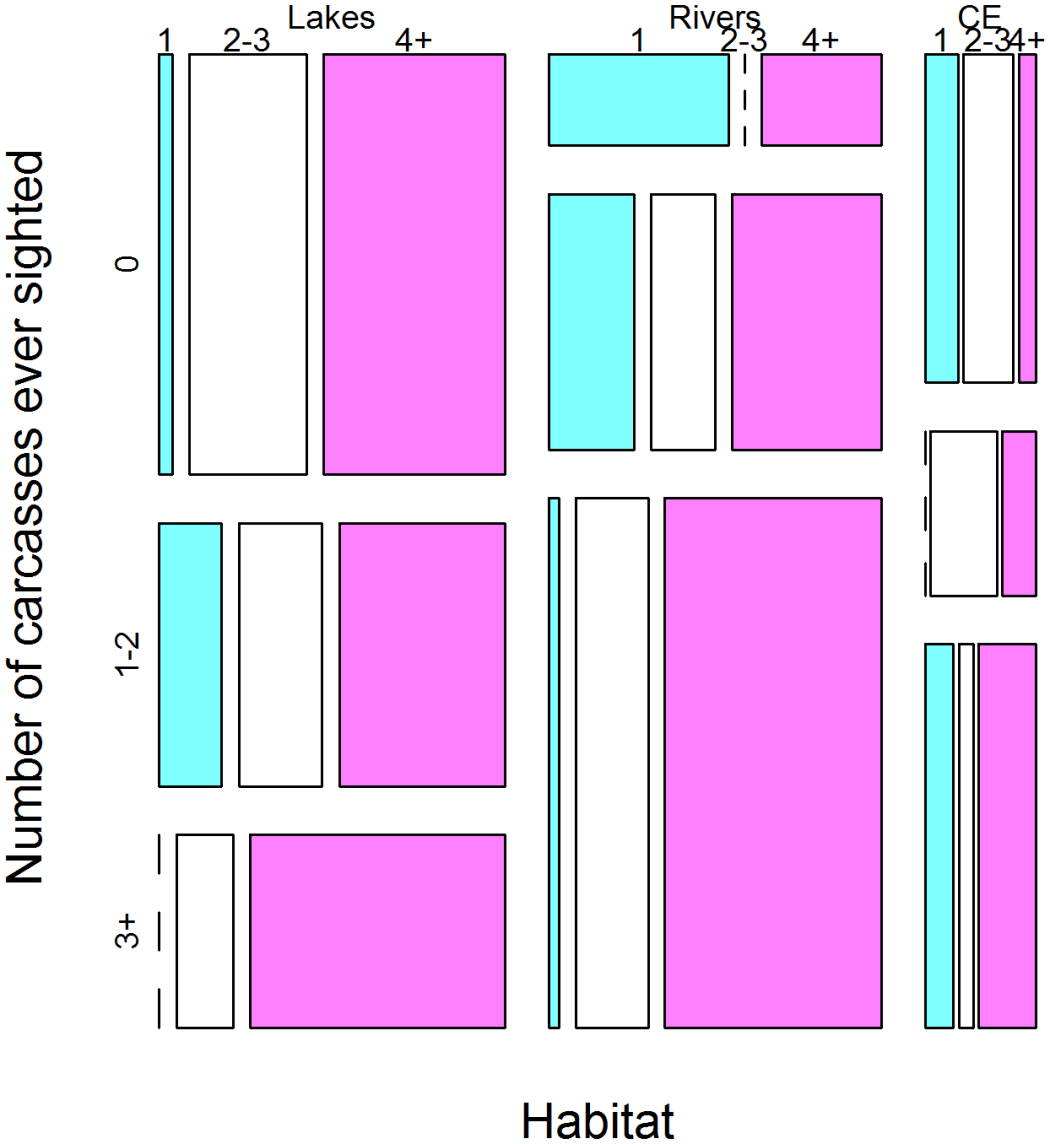
Mosaic plot of patterns in the sightings of dead manatees. The number of dead manatees ever sighted, D (0, 1–2, and 3+) reveals two patterns. Firstly, the sighting of three manatee carcasses or more (D3+) is strongly associated with the sighting of four live manatees or more (L4+). Secondly, the lower reaches of the rivers and the coast & estuaries are highly associated with the sighting of more carcasses.

The sighting of three dead manatees or more (D 3+) associated strongly with the sighting of four live manatees or more (L 4+), regardless of habitat (${\hat{\mu }}_{3+,4+}^{D∙L}\;=\;1.858\pm 0.7767,\;Z\;=\;2.393,\;p<0.05$). Of all three habitat types, the rivers are more conducive for sighting large number of manatee carcasses, precisely three or more (${\hat{\mu }}_{riv,\;3+}^{H∙D}\;=\;2.591\pm 0.600,\;Z\;=\;4.317,\;p<0.001$) and one or two (${\hat{\mu }}_{riv,1-2}^{H∙D}\;=\;1.388\pm 0.595,\;Z\;=\;2.334,\;p<0.05$). These patterns are obvious from the mosaic plot (Fig 4) and the model output in Table 4.

**Table 4:** Fitted log-linear Poisson model for patterns in the sightings of dead manatees.

Model effects	Estimates	SE	Z-statistics
Intercept	1.000	0.5286	1.893
Rivers	-0.402	0.6942	-0.579
Coast & estuaries	-0.067	0.7057	-0.095
Dead (1–2)	-0.416	0.6454	-0.644
Dead (3+)	-2.274	0.8008	-2.840**
Live (2–3)	1.163	0.5969	1.948^†^
Live (4+)	1.628	0.5706	2.853**
Rivers x Dead (1–2)	1.388	0.5947	2.334*
Coast & estuaries x Dead (1–2)	-0.144	0.7346	-0.197
Rivers x Dead (3+)	2.591	0.6001	4.317***
Coast & estuaries x Dead (3+)	1.313	0.6737	1.948^†^
Rivers x Live (2–3)	-1.308	0.7426	-1.761^†^
Coast & estuaries x Live (2–3)	-1.075	0.8019	-1.340
Rivers x Live (4+)	-1.313	0.6875	-1.909^†^
Coast & estuaries x Live (4+)	-2.021	0.8115	-2.490*
Dead (1–2) x Live (2–3)	-0.136	0.7073	-0.192
Dead (3+) x Live (2–3)	0.988	0.8203	1.204
D(1–2) x L(4+)	0.021	0.6718	0.031
D(3+) x L(4+)	1.858	0.7767	2.393*
**Fit statistics** ^‡^	**AIC**: NONE		
	**Null deviance**: 113.72(26 df)		
	**Res. deviance**: 14.87(8 df)		

The parameter estimates, standard errors, Wald’s Z-statistics and fit statistics were obtained using a penalized (reduced-bias) maximum likelihood (see details in Firth [57] and Kosmidis and Firth [58]).

^†^,*,**,*** significant at probability levels of 10%, 5%, 1%, and 0.1%, respectively.

^‡^ The R script used in implementing the penalized maximum likelihood does not return a value for the Akaike’s Information Criterion (AIC).

**Fig 5:**
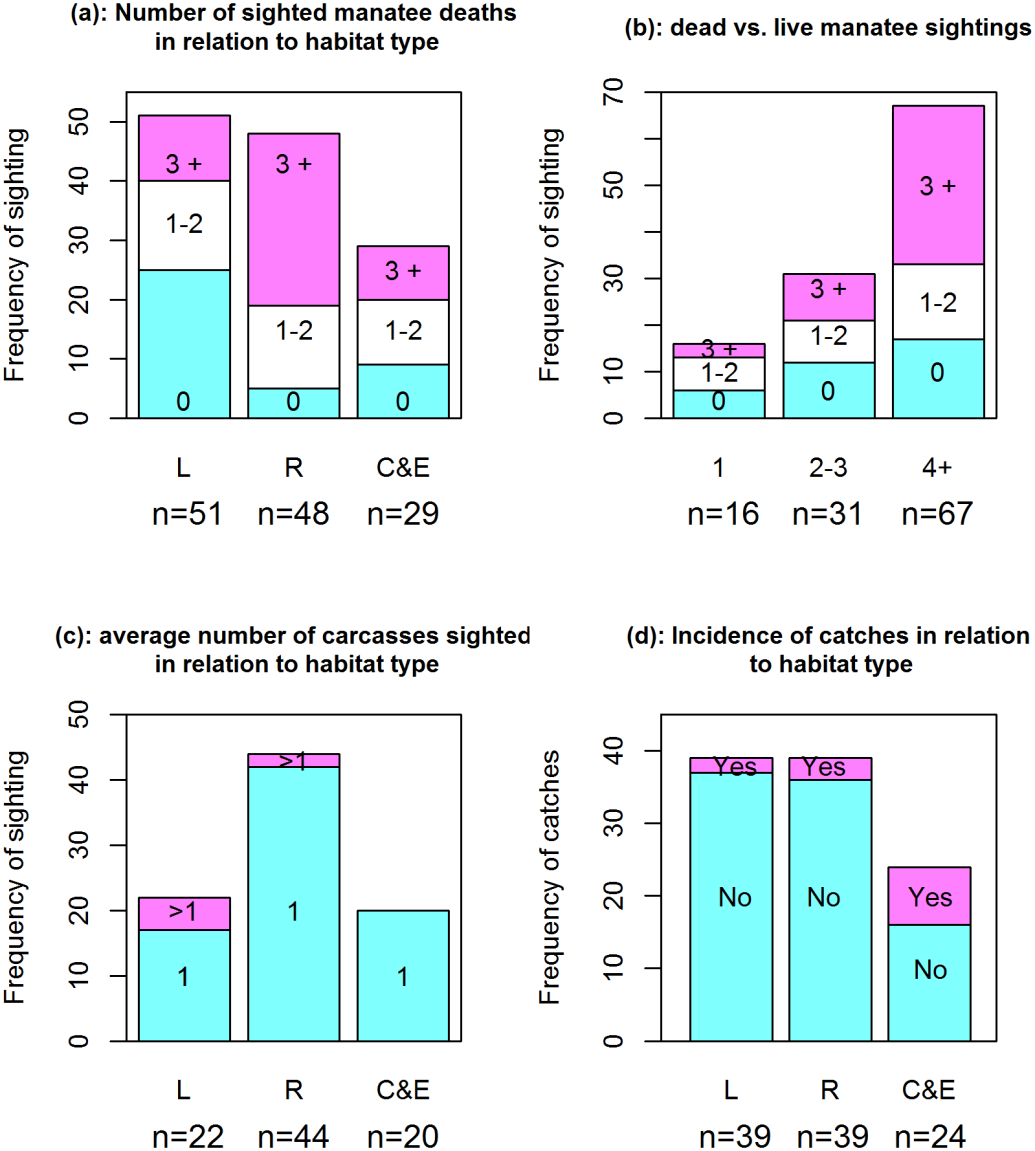
Reported incidence of manatee mortality in lakes (L), rivers (R), and coast & estuaries (C&E). The main features are that: (a) the number of sighted manatee carcasses increases downstream the lakes (with a peak in the rivers); (b) sighting three manatee carcasses or more associates highly with the sighting of four live manatees or more; (c) on average, one manatee carcass is sighted in all habitat types; however, such sightings are more frequent in the lakes and, to a lesser extent, in the rivers; (d) the incidence of catches increases from the lakes to the rivers and then to the coast & estuaries.

The association between the type of habitat and the number of sighted carcasses comes out clearly in Fig 5a, while the association between the frequencies of live and dead manatees is most obvious in Fig 5b. Fig 5c clearly conveys that single carcasses are most likely to be sighted in all habitat types; only, such sightings are more frequent in the rivers, compared to either the lakes or the coast & estuaries.

Fishers proffered several combinations of causes to account for manatee deaths: hunting (52 respondents out of 82), food intoxication (12 respondents), net strangulation (9 respondents), old age (4 respondents) and boat collision (1 respondent). Incidental catches were further assessed by asking fishers whether their net has ever caught a manatee. The log odds of the probability of an incidental catch deviated unequivocally from zero in the lakes $\text{(}{\hat{\beta }}_{0}\;=\;-2.92\pm 0.73\text{)}$ and the coast & estuaries $({\hat{\beta }}_{2}=\text{ 2.22}\pm \text{ 0.85)}$ but not in the rivers $({\hat{\beta }}_{1}=\text{ 0.43}\pm \text{ 0.94)}$ (Table 3 and Fig 5d). Actually, the odds of an incidental catch increased from the lakes (0.054) to the coast & estuaries (0.50) through the rivers (0.083).

### Perceived trend in manatee numbers

Finally, the interviewees were further requested to say whether the trend in manatee numbers was constant, increasing or decreasing. Pooling the first two categories, a significant habitat effect emerged whereby (see Table 3 and Fig 3d), the log-odds of a perceived increasingor stable trend in manatee numbers is maximum in the lakes $\text{(}{\hat{\beta }}_{0}\;=\;3.09\pm 0.72\text{)}$ and declines progressively as one moves down to the rivers $({\hat{\beta }}_{1}=\text{-1.77 }\pm \text{0.83)}$ and ultimately to the coast & estuaries $({\hat{\beta }}_{3}=\text{-2.22 }\pm \text{0.90).}$ Alternatively, the odds of a perceived increasing or stable trend in manatee numbers declined precipitously from 22.0 (in the lakes) to 3.75 (in the rivers) and 2.40 (at the coast & estuaries).

Of the 81 respondents saying that manatee numbers were increasing, 57 attributed that trend to reduced hunting pressure, as against 20 to high reproduction rate, and 8 without opinion. All but one of the 13 respondents who perceived a negative trend in manatee numbers blamed it on hunting pressure (6 respondents) and/or reduced water level (7 respondents).

## Discussion

### Patterns in the sightings of live manatees

The high proportion of fishers regularly sighting manatees across all habitat types corroborates earlier accounts on the species presence in the study area [4, 6, 7]. Furthermore, six out of ten interviewees (58.5±9%) declared sighting manatees four or more times a month, i.e. averagely once a week, which compares well with other parts of Africa (Keith Diagne, unpublished data).

This study identified two possible manatee hotspots: one in Lake Ossa and another in the Malimba-Mbiako section of the River Sanaga, towards the estuary (the red circles in Fig 1). The reported odds of live manatee sightings decreased significantly from the lakes to the rivers, and further towards the estuaries and the Atlantic coast (Table 3). Furthermore, the sighting of live manatees occurred frequently in the rivers, during the evenings of the rainy seasons, presumably when water levels are high and the river bottom muddier, thus allowing benthic activities.

These occurrence patterns elicit two comments. Firstly, manatees inhabit practically every area accessible to them in coastal wetlands *viz*., rivers, estuaries, marshes, and inlets and occasionally the sea [6, 8, 12], insofar as floating freshwater plants and tree cover are available along aquatic systems [6, 13]. Secondly, the emerging pattern in manatee occurrence is consistent with the species dependence on a regular access to freshwater [59]. After foraging on marine seagrasses, Florida manatees would travel inland to access fresh water about once a week [60]. Similar movements are not unlikely in our study area, as access is open between fresh water sources and marine seagrass beds along the coast and in the estuaries.

The output of the log-linear Poisson model further suggested that manatees would have a diurnal activity pattern in the lakes (especially in the dry season) as opposed to a crepuscular to nocturnal activity pattern in the rivers (especially in the wet season). Therefore, we hypothesise that Lake Ossa and other deep pools provide manatees with dry-season refuge and perhaps year round sanctuary. In the wet season, manatees move into River Sanaga through a small canal (from fishers’ experience), aided most probably by higher water levels and muddier bottom. The crepuscular or nocturnal activity patterns in the rivers may be an adaptive response to increased boat traffic, human settlements and hunting pressure [6, 9, 45–47, 61].

The manatee is a mildly social species, for which singletons and dyads are sighted more often than larger groups [8, 9, 40]. However, Florida manatees tend to aggregate at warm water sites in winter, at resource-concentration sites, and on females in oestrous during mating seasons [62–63]. Kouadio [9] also reported larger group sizes at lower temperatures for West African manatees, although the correlation coefficient he quoted was spurious due to a very large sample size. Manatees are intolerant of temperatures below 20°C [64], a threshold that triggers annual migrations to warmer water sources in Florida manatees [62]. This required minimum temperature is exceeded in the tropics; in Lake Ossa, for instance, the temperature range is 28.5°C–33.2°C (Paul R. Ngafack, unpublished data). Thus, the large group sizes reported by fishers might actually have referred to distinct sightings made at close space or time interval, rather than genuine clusters, notwithstanding possible increase in group size during colder months (i.e. August to September) or mating periods.

The availability of food, thermal, and fresh water resources determine habitat selection by (Florida) manatees. However, for females with dependent calves, three factors are of critical importance: low ambient noise, to enable auditory contact between mothers and calves; absence of currents, to ease the movement of mother-calf pairs; and increased foraging requirements, due to the increase of metabolism associated with nursing a calf [65]. Manatees are large, opportunistic herbivores that require a high biomass of aquatic macrophytes to sustain their nutritional needs [1, 6, 9, 66], notwithstanding the occasional carnivory [67–68]. Therefore, it can be reasonably conjectured that manatees would shift to the consumption of mangrove (and perhaps detritus) materials when the ecosystem is poor, i.e. has a low biomass of primary producers: detritus, mangroves, aquatic autotrophs, and phytoplankton [66] or, alternatively, when forage amount and quality decline in the late dry season. In either case, efforts are needed to curb the high rate at which the mangroves of West-Central Africa are currently vanishing, i.e. between 0.5 and 0.7 million of ha since 1985 [69–70]. In the same vein, we recommend protecting the habitat types used by females with dependent calves, bearing in mind that the birth season in African and Amazonian manatees coincides with the period of high plant productivity [1, 6] which is in the wet season.

It is not clear whether the habitat systems defined for the Antillean manatee [71] holds true either entirely or partially for the West African manatee. It is also not clear which categories in that typology are essential areas, as the concept of essential area is still being debated [34]. In any case, several seasonally fluctuating factors have been shown to affect the occurrence, activity, and movements of (West African) manatees. These factors include:

1. water temperature: warm-water refugia are necessary for manatee survival as the species is intolerant of temperatures below 20°C [62, 64–65];
2. water current: manatee energetic expenditures increase with higher currents [6, 13, 65], which is most likely in rainy season, though critical limits are unknown;
3. water salinity: Florida manatees that inhabit marine environments require regular access to fresh water sources [59–60]and there is also evidence that those living in freshwater systems require some access to sodium [59]; hence, manatee movements are closely tied to seasonal changes in fresh and salt water flows, whether along river streams or between lagoons and the sea [9, 72];
4. water levels: the high depths in wet season facilitate escape from intruders, access to bank vegetation, and movements to flooded forests and swamps [6, 9] but encounters are more likely at sites with 2–5 m depth [9, 13, 71, 73];
5. bottom substrate: manatees prefer muddy substrates which, unlike rocky ones, provide resting holes and allow benthic activities e.g., gliding and rooting [12, 14, 73].

The above (loosely connected) statements do not yet form a theory as philosophers of science understand it (see, e.g. [74]). Rather, they provide a useful set of semi-theoretical results, from which hypotheses can be formulated about manatee distribution, habitat use, and movements [6, 34, 40]. In particular, we hypothesise that manatee occurrence is patterned after the following spatio-temporal gradient in sighting frequency: high in the lakes and other deep pools (year round); fair downstream the lakes, at sites where rivers are wide, deep, with a gentle current flow such as bends, cutoffs, and coves (in both seasons); and low further downstream *viz*. in the estuaries and along the Atlantic coast (in wet season). While consistent with our findings (to some extent), the conjectured pattern in manatee occurrence needs to be confirmed or falsified by future research field work using sonar and radio-telemetry technology. It will be equally interesting to know which of the above statements explains the manatee hotspots identified by fishers.

### Patterns in the sightings of dead manatees

The odds of reported incidental catches increased from the lakes to the rivers and ultimately to the coast & estuaries (Table 4). In effect, the fitted log-linear Poisson model for sighting manatee carcasses unveiled two distinct patterns (Table 4). On the one hand, the sighting of three manatee carcasses or more is highly associated with the sighting of four live manatees or more (Fig 5b). On the other hand, the lower reaches of the rivers associated strongly with the sighting of more manatee carcasses, precisely three carcasses or more (Fig 5a).

There are three possible explanations to these sighting patterns. Firstly, everything else being equal, the number of deaths normally would be higher where the population is larger (e.g., in the Malimba-Mbiako section of River Sanaga, one of the two identified manatee hotspots). Secondly, the carcasses sighted in the lower reaches are adrift from the upper reaches where, arguably, the mortality incidence is at least similar if not higher, although the reported incidence of catches was clearly lowest in the lakes (see further below). Thirdly, the perceived mortality pattern may derive from experienced fishers being equally good at sighting live and dead manatees, per the model output just mentioned. This issue can be settled empirically through a sound monitoring program for collecting data on manatee presence, abundance, and distribution [50–52, 75] but also, granted a standardized necropsy protocol [76], data on manatee mortality factors, reproduction status, diet, and body condition, amongst others. We further hypothesize that excess mortality rate in the rivers may be due to an increased risk of stranding and illegal killing when manatees attempt to collect their food on flooded forest and river banks in the rainy season [6,44].

### Perceived trends in manatee numbers

Despite directed and incidental catches, the number of manatees was perceived as increasing or stable, thus corroborating earlier assessments that the study area sustains a considerable manatee population [4, 6, 7]. In particular, the odds of a perceived increasing or stable trend in manatee numbers are highest in the lakes whence it plummets in the rivers, estuaries, and Atlantic coast (Table 3).

Some interviewees explained the growing number of manatees by a high reproduction rate. This argument is supported neither by the demographics nor the life history parameters that are available for other manatee species. In effect, manatees are long-lived species: the maximum life expectance is 60 years and the average first age of reproduction is 5 years in females [77]. They tend to reproduce slowly due to a gestation period of 12–14 months, a calving interval of 2.5 years, and a tendency for delaying reproduction in adverse conditions [1].

As evidence for increasing manatee numbers, fishers (also in Korup National Park, Pascal Koh-Dimbot pers. comm.) mention the growing incidence of torn nets which, for us, reflects the growing fishing pressure. In effect, as nylon and polyester gillnets are increasingly set across waterways and other sensitive locations such as rivers mouths, they are difficult to avoid by manatees [9, 77], thus increasing the odds of incidental catches. Cases of manatee by-catch have also been reported for hook-line gear in the Comoros and Cameroon [22]. Despite the dearth of demographic data (population size, birth and mortality rates, and population distribution) in the Lower Sanaga Basin, we posit that there may actually be several manatee populations which vary in their size locality-wise in relation to the intensity of human activities. Further, the dynamics of metapopulations (see [78]: pp.104-108) can be invoked to explain manatee persistence in the Lower Sanaga Basin. But even so, a recent model output has suggested that the dynamics of a metapopulation may not be able to withstand an annual human-induced mortality rate above 5% [79].

### Implications for data requirements

Survey questionnaires are useful in studying Sirenian populations and their habitats [13, 80]. The reference habitats in this study included lakes, rivers, estuaries, and the Atlantic coast, which all have easily identifiable boundaries. However, we realize that fishers do not ply their trade with equal intensity throughout the study zone and certainly not over seasons, the wet season being a slack period relatively to the dry season.

This heterogeneous intensity in manatee sighting over space and time is liable to affect the bias and efficiency of statistical estimates. The statistical estimates reported in this study pertain each to a specific habitat type, as we never envisaged an average of them over habitat types. Because fishers only visit small areas within each of the reference habitats, there is a real concern about possible bias (or systematic error) in the estimates reported. Bias, however, is not an issue insofar as the areas visited collectively by the surveyed fishers actually form a representative sample of key components in each reference habitat. Furthermore, the estimators of proportions and model parameters are unbiased and consistent [54–55, 81], under general conditions that were met in this study.

Ecologically, the representativeness of habitats relates closely to the sampling scale. The small scale at which fishers operate defines the so-called within (or alpha) habitat diversity [82] which obviously is not appropriate for the conservation of manatee populations. To be effective, the latter must be envisaged preferably at a regional scale that encompasses a mosaic of habitats across state boundaries, in extent of several hundreds of kilometers. At this multinational scope, however, in-country constraints are likely to be compounded by cooperation difficulties. Therefore, it would be more realistic to consider an intermediate spatial scale that covers more than one habitat or community, also known as between (or beta) habitat diversity, as a first step towards a regional integration of management and conservation strategies [16, 42–43].

In order to palliate the above shortcomings of survey questionnaires, we recommend setting up at intermediate to regional scales, a “participatory” ecological monitoring program aimed at tracking space and time trends in manatee population abundance, structure and distribution, amongst other state variables [50–52], leading to the possible development of a stochastic population dynamics model. This recommendation is consonant with the 12 Malawi principles for the ecosystem approach to management, as defined in the Convention of Biological Diversity (www.cbd.int/ecosystem, see also [83]). These principles are complementary and apply as a whole. However, two of them are particularly relevant to our recommendation: the identification of an appropriate spatial scale (principle 7) and the integration of scientific and indigenous and local knowledge (principle 11). Accordingly, the recommended monitoring program would imply that, at the very least, the study area is divided into sectors and a rational methodology is set up to collate information that fishers would gather on manatees during their regular outings. Two worthy program components might concern a standardised necropsy protocol for collecting mortality and biological data together with sonar and radio-telemetry technology to discern manatee use and movements between critical habitat components.

## Supporting Information

S1 Fig Mosaic plot of live manatee sighting patterns obtained with fitted cell values. (DOCX)

S2 Fig Mosaic plot of dead manatee sighting patterns obtained with fitted cell values. (DOCX)

S1 Table Manatee sighting and occurrence patterns. The table gives the distribution of respondents for the questionnaire items: manatee sighting, monthly number of sighting, maximum number of manatees sighted on a single occasion, and sighting context (habitat, season, and time of day).
(DOCX)

S2 Table Patterns in live manatee sighting. This is the complete three-way contingency table of manatee sighting frequencies by time of day (TOD), season and habitat. The mosaic plot (Fig 2) and the fitted log-linear Poisson model (Table 2) were both obtained from this table after omitting the first level of TOD, viz. “Anytime”, and the first level of Season, viz. “Both” (in an attempt to reduce data sparseness, i.e. the number of small cells).
(DOCX)

S3 Table ANOVA of the fitted log-linear Poisson model for live manatee sighting patterns. The effects tested correspond to the fits in Table 2 and were tested using the likelihood ratio chi-squared statistic.
(DOCX)

S4 Table The fitted cells values obtained with the model for live manatee sighting patterns. (DOCX)

S5 Table Patterns in dead manatee sighting. Three-way contingency table of the frequency of dead manatee sighting (D) crossed by habitat (H), and the number of live manatees sighted at once (L). The mosaic plot (Fig 4) and the fitted log-linear Poisson model (Table 4) were obtained using this table.
(DOCX)

S6 Table ANOVA of the fitted log-linear Poisson model for dead manatee sighting patterns. The effects correspond to the fits in Table 4 and were tested using the likelihood chi-squared statistic.
(DOCX)

S7 Table The fitted cells values obtained with the model for dead manatee sighting patterns. (DOCX)

S8 Table Sighted manatee deaths and perceived trend in manatee numbers. The table gives the distribution of respondents for the questionnaire items: number of manatee deaths sighted, number of dead manatees sighted at once, and the perceived trend in manatee numbers.
(DOCX)
