# Supplementary material for: Using Pooled Local Expert Opinions (PLEO) to Discern Patterns in Sightings of Live and Dead Manatees (Trichechus senegalensis, Link 1785) in Lower Sanaga Basin, Cameroon
Source: PLoS One. 2015 Jul 21;10(7):e0128579. doi: 10.1371/journal.pone.0128579 (PMC4511414; doi:10.1371/journal.pone.0128579)
Supplement: S2 Fig — (DOCX) [file pone.0128579.s002.docx]

**S2 Fig.** **Mosaic plot of dead manatee sighting patterns obtained with fitted cell values.**
