# Supplementary material for: Using Pooled Local Expert Opinions (PLEO) to Discern Patterns in Sightings of Live and Dead Manatees (Trichechus senegalensis, Link 1785) in Lower Sanaga Basin, Cameroon
Source: PLoS One. 2015 Jul 21;10(7):e0128579. doi: 10.1371/journal.pone.0128579 (PMC4511414; doi:10.1371/journal.pone.0128579)
Supplement: S1 Table — The table gives the distribution of respondents for the questionnaire items: manatee sighting, monthly number of sighting, maximum number of manatees sighted on a single occasion, and sighting context (habitat, season, and time of day). (DOCX) [file pone.0128579.s003.docx]

**S1 Table. Manatee sighting and occurrence patterns.**

| **Question (item)** | **Answers** | **Habitat type** | | | **Chi squared test**^†^ | |
| --- | --- | --- | --- | --- | --- | --- |
|  |  | **Coast & Estuary** | **Lakes** | **Rivers** | Method | $\chi^{2}(df)$ |
| 1-Have you ever sighted a manatee? | Yes | 7 | 35 | 25 | Pearson | 13.3(2)^**^ |
|  | No | 23 | 19 | 25 |  |  |
| 2-How often do you see manatees in a month? | 1 | 4 | 4 | 9 | Pearson | 6.4(4) |
|  | 2-3 | 6 | 16 | 10 |  |  |
|  | 4 + | 6 | 32 | 30 |  |  |
| 3-How many manatees do you use to sight most at once? | 1 manatee | 3 | 4 | 7 | Pearson | 1.6(4) |
|  | 2-3 manatees | 16 | 22 | 27 |  |  |
|  | 4 manatees or more | 11 | 11 | 11 |  |  |
| 4-During which season do you sight manatee most? | Rainy season | 13 | 21 | 30 | LRT | 11.5(4)^*^ |
|  | Dry sea season | 10 | 30 | 12 |  |  |
|  | Both | 4 | 3 | 7 |  |  |
| 5-At what time of the day do you see them often? | Morning (0500-1100 hours) | 6 | 15 | 2 | LRT | 36.5(6)^***^ |
|  | Midday/afternoon(1100-1800 hours) | 2 | 11 | 1 |  |  |
|  | Evening (1800-2100) | 7 | 7 | 28 |  |  |
|  | Anytime | 13 | 18 | 18 |  |  |
| 6-At which water level do you sight manatees most? | Low water level | 14 | 13 | 4 | LRT | 21.1(2)^***^ |
|  | High water level | 5 | 9 | 20 |  |  |
|  | Both | 9 | 3 | 12 |  |  |

The table gives the distribution of respondents for the questionnaire items: manatee sighting, monthly number of sighting, maximum number of manatees sighted on a single occasion, and sighting context (habitat, season, and time of day).

^†^ The test used was either the Pearson’s chi-square or its likelihood ratio test (LRT) approximation.

^*^, ^***^: Significant at probability levels 0.05 and 0.001, respectively.
