# Supplementary material for: Using Pooled Local Expert Opinions (PLEO) to Discern Patterns in Sightings of Live and Dead Manatees (Trichechus senegalensis, Link 1785) in Lower Sanaga Basin, Cameroon
Source: PLoS One. 2015 Jul 21;10(7):e0128579. doi: 10.1371/journal.pone.0128579 (PMC4511414; doi:10.1371/journal.pone.0128579)
Supplement: S2 Table — This is the complete three-way contingency table of manatee sighting frequencies by time of day (TOD), season and habitat. The mosaic plot (Fig 2) and the fitted log-linear Poisson model (Table 2) were both obtained from this table after omitting the first level of TOD, viz. “Anytime”, and the first level of Season, viz. “Both” (in an attempt to reduce data sparseness, i.e. the number of small cells). (DOCX) [file pone.0128579.s004.docx]

**S2 Table.** **Patterns in live manatee sighting.**

| Habitat (H) | Season (S) | Time of day (TOD) | | |  |
| --- | --- | --- | --- | --- | --- |
|  |  | Anytime | Morning | Midday/  Afternoon | Evening |
| Lakes | Both | 1 | 0 | 1 | 0 |
|  | Dry | 5 | 5 | 6 | 1 |
|  | Rainy | 5 | 6 | 2 | 2 |
|  |  |  |  |  |  |
| Rivers | Both | 0 | 0 | 0 | 2 |
|  | Dry | 3 | 0 | 0 | 3 |
|  | Rainy | 5 | 0 | 1 | 11 |
| Coast & estuary |  |  |  |  |  |
|  | Both | 1 | 0 | 0 | 1 |
|  | Dry | 1 | 1 | 0 | 0 |
|  | Rainy | 1 | 1 | 0 | 1 |
|  |  |  |  |  |  |

This is the complete three-way contingency table of manatee sighting frequencies by time of day (TOD), season and habitat. The mosaic plot (Fig. 2) and the fitted log-linear Poisson model (Table 2) were both obtained from this table after omitting the first level of TOD, viz. “Anytime”, and the first level of Season, viz. “Both” (in an attempt to reduce data sparseness, i.e. the number of small cells).
