# Supplementary material for: Using Pooled Local Expert Opinions (PLEO) to Discern Patterns in Sightings of Live and Dead Manatees (Trichechus senegalensis, Link 1785) in Lower Sanaga Basin, Cameroon
Source: PLoS One. 2015 Jul 21;10(7):e0128579. doi: 10.1371/journal.pone.0128579 (PMC4511414; doi:10.1371/journal.pone.0128579)
Supplement: S3 Table — The effects tested correspond to the fits in Table 2 and were tested using the likelihood ratio chi-squared statistic. (DOCX) [file pone.0128579.s005.docx]

**S3 Table.** **ANOVA of the fitted log-linear Poisson model for live manatee sighting patterns.**

| Term | Effect | |  | Residual | |  | Prob^†^ |
| --- | --- | --- | --- | --- | --- | --- | --- |
|  | df | Deviance |  | Df | Deviance |  |  |
| Null |  |  |  | 17 | 60.11 |  |  |
| Habitat | 2 | 16.62 |  | 15 | 43.49 |  | 0.001 |
| Season | 1 | 1.61 |  | 14 | 41.88 |  | 0.204 |
| TOD | 2 | 3.07 |  | 12 | 38.81 |  | 0.215 |
| Habitat x Season | 4 | 30.26 |  | 8 | 8.54 |  | < 10^-6^ |
| Season x TOD | 2 | 5.37 |  | 6 | 3.18 |  | 0.017 |

The effects tested correspond to the fits in Table 2 and were tested using the likelihood ratio chi-squared statistic.

^†^ Probability of larger log likelihood chi-squared statistic.
