# Supplementary material for: Using Pooled Local Expert Opinions (PLEO) to Discern Patterns in Sightings of Live and Dead Manatees (Trichechus senegalensis, Link 1785) in Lower Sanaga Basin, Cameroon
Source: PLoS One. 2015 Jul 21;10(7):e0128579. doi: 10.1371/journal.pone.0128579 (PMC4511414; doi:10.1371/journal.pone.0128579)
Supplement: S4 Table — (DOCX) [file pone.0128579.s006.docx]

**S4 Table. The fitted cells values obtained with the model for live manatee sighting patterns**.

| Season=Dry | | | |
| --- | --- | --- | --- |
| TOD | | | |
| Habitat | Morning | Midday/  Afternoon | Evening |
| Lakes | 5.5234 | 5.7881 | 0.8502 |
| Rivers | 0.2401 | 1.0214 | 3.5222 |
| CE | 1.2007 | 0.3405 | 0.3644 |
| Season=Rainy | | | |
| TOD | | | |
| Habitat | Morning | Midday/  Afternoon | Evening |
| Lakes | 6.3732 | 3.1167 | 2.7395 |
| Rivers | 0.2771 | 0.5500 | 11.3495 |
| CE | 1.3855 | 0.1833 | 1.1741 |
