# Supplementary material for: Using Pooled Local Expert Opinions (PLEO) to Discern Patterns in Sightings of Live and Dead Manatees (Trichechus senegalensis, Link 1785) in Lower Sanaga Basin, Cameroon
Source: PLoS One. 2015 Jul 21;10(7):e0128579. doi: 10.1371/journal.pone.0128579 (PMC4511414; doi:10.1371/journal.pone.0128579)
Supplement: S5 Table — Three-way contingency table of the frequency of dead manatee sighting (D) crossed by habitat (H), and the number of live manatees sighted at once (L). The mosaic plot (Fig 4) and the fitted log-linear Poisson model (Table 4) were obtained using this table. (DOCX) [file pone.0128579.s007.docx]

**S5 Table. Patterns in dead manatee sighting.**

| Habitat (H) | Frequency of dead manatee sighting (D) | Group size of sighted live manatees (L) | | |  |
| --- | --- | --- | --- | --- | --- |
|  |  | 1 | 2-3 | 4+ | |
| Lakes | Never | 1 | 9 | 14 | |
|  | Once/twice | 3 | 4 | 8 | |
|  | Thrice + | 0 | 2 | 9 | |
|  |  |  |  |  | |
| Rivers | Never | 3 | 0 | 2 | |
|  | Once/twice | 4 | 3 | 7 | |
|  | Thrice + | 1 | 7 | 21 | |
| Coast & estuary |  |  |  |  | |
|  | Never | 2 | 3 | 1 | |
|  | Once/twice | 0 | 2 | 1 | |
|  | Thrice + | 2 | 1 | 4 | |
|  |  |  |  |  | |

Three-way contingency table of the frequency of dead manatee sighting (D) crossed by habitat (H), and the number of live manatees sighted at once (L). The mosaic plot (Fig. 4) and the fitted log-linear Poisson model (Table 4) were obtained using this table.
