# Supplementary material for: Using Pooled Local Expert Opinions (PLEO) to Discern Patterns in Sightings of Live and Dead Manatees (Trichechus senegalensis, Link 1785) in Lower Sanaga Basin, Cameroon
Source: PLoS One. 2015 Jul 21;10(7):e0128579. doi: 10.1371/journal.pone.0128579 (PMC4511414; doi:10.1371/journal.pone.0128579)
Supplement: S6 Table — The effects correspond to the fits in Table 4 and were tested using the likelihood chi-squared statistic. (DOCX) [file pone.0128579.s008.docx]

**S6 Table.** **ANOVA of the fitted log-linear Poisson model for dead manatee sighting patterns.**

| Term | Effect | |  | Residual | |  | Prob^†^ |
| --- | --- | --- | --- | --- | --- | --- | --- |
|  | df | Deviance |  | Df | Deviance |  |  |
| Null |  |  |  | 26 | 113.718 |  |  |
| Habitat | 2 | 22.191 |  | 24 | 91.528 |  | <0.0001 |
| Dead | 2 | 3.226 |  | 22 | 88.302 |  | 0.20 |
| Live | 2 | 35.689 |  | 20 | 52.613 |  | <0.0001 |
| Habitat*Dead | 4 | 22.866 |  | 16 | 29.747 |  | <0.001 |
| Habitat*L | 4 | 5.960 |  | 12 | 23.787 |  | 0.20 |
| Dead*Live | 4 | 10.086 |  | 8 | 13.701 |  | 0.039 |

The effects correspond to the fits in Table 4 and were tested using the likelihood chi-squared statistic.

^†^ Probability of larger likelihood chi-square statistic.
