# Supplementary material for: Using Pooled Local Expert Opinions (PLEO) to Discern Patterns in Sightings of Live and Dead Manatees (Trichechus senegalensis, Link 1785) in Lower Sanaga Basin, Cameroon
Source: PLoS One. 2015 Jul 21;10(7):e0128579. doi: 10.1371/journal.pone.0128579 (PMC4511414; doi:10.1371/journal.pone.0128579)
Supplement: S7 Table — (DOCX) [file pone.0128579.s009.docx]

**S7 Table. The fitted cells values obtained with the model for dead manatee sighting patterns.**

| Number of dead manatees =0 | | | |
| --- | --- | --- | --- |
| Number of live manatees | | | |
| Habitat | 1 | 2-3 | 4+ |
| Lakes | 2.7194 | 8.7018 | 13.8542 |
| Rivers | 1.8196 | 1.5745 | 2.4948 |
| CE | 2.5434 | 2.7784 | 1.7176 |

| Number of dead manatees =1-2 | | | |
| --- | --- | --- | --- |
| Number of live manatees | | | |
| Habitat | 1 | 2-3 | 4+ |
| Lakes | 1.7947 | 5.0129 | 9.3360 |
| Rivers | 4.8121 | 3.6344 | 6.7364 |
| CE | 1.4529 | 1.3854 | 1.002 |

| Number of dead manatees =3+ | | | |
| --- | --- | --- | --- |
| Number of live manatees | | | |
| Habitat | 1 | 2-3 | 4+ |
| Lakes | 0.2798 | 2.4044 | 9.1410 |
| Rivers | 2.4983 | 5.8052 | 21.9643 |
| CE | 0.9724 | 2.8528 | 4.2111 |
