# Supplementary material for: Using Pooled Local Expert Opinions (PLEO) to Discern Patterns in Sightings of Live and Dead Manatees (Trichechus senegalensis, Link 1785) in Lower Sanaga Basin, Cameroon
Source: PLoS One. 2015 Jul 21;10(7):e0128579. doi: 10.1371/journal.pone.0128579 (PMC4511414; doi:10.1371/journal.pone.0128579)
Supplement: S8 Table — The table gives the distribution of respondents for the questionnaire items: number of manatee deaths sighted, number of dead manatees sighted at once, and the perceived trend in manatee numbers. (DOCX) [file pone.0128579.s010.docx]

**S8 Table.**  **Sighted manatee deaths and perceived trend in manatee numbers**.

| **Question (item)** | **Answers** | **Habitat type** | | | **Chi squared test**^†^ | |
| --- | --- | --- | --- | --- | --- | --- |
|  |  | **Coast & Estuary** | **Lakes** | **Rivers** | Method | $\chi^{2}(df)$ |
| 7-How many times have you ever seen a dead manatee? | Never | 9 | 25 | 5 | LRT | 24.0(4)^***^ |
|  | Once to thrice | 11 | 15 | 14 |  |  |
|  | More than thrice | 9 | 11 | 29 |  |  |
| 8-How many dead manatees do you often sight at once? | Only one | 0 | 5 | 2 | LRT | 8.7(2)^*^ |
|  | More than one | 20 | 17 | 42 |  |  |
| 9-How do manatee populations change in your area? | Increase or constant | 12 | 44 | 30 | LRT | 8.7(2)^*^ |
|  | Decrease | 5 | 2 | 8 |  |  |

The table gives the distribution of respondents for the questionnaire items: number of manatee deaths sighted, number of dead manatees sighted at once, and the perceived trend in manatee numbers.

^†^ The test used was either the Pearson’s chi-square or its likelihood ratio test (LRT) approximation.

^*^, ^***^: Significant at probability levels 0.05 and 0.001, respectively.
